# Safer ocular anaesthesia

**Published:** 2016

**Authors:** Tom Eke

**Affiliations:** Consultant Ophthalmologist: Norfolk & Norwich University Hospital, UK **tom.eke@nnuh.nhs.uk**

**Figure:**
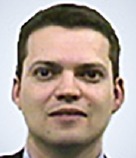
Tom Eke

Local anaesthesia for eye surgery is usually safe, but serious complications may sometimes occur. Sharp-needle blocks (retrobulbar or peribulbar) can potentially cause blindness or even death. Sub-Tenon's blocks, using a blunt-ended cannula, appear to be much safer. However, they can also cause serious complications.

## Sharp-needle techniques (retrobulbar and peribulbar injections)

These appear to have the highest likelihood of serious complications. Blindness may occur if the needle pierces the globe or optic nerve. If the local anaesthetic is inadvertently injected inside the optic nerve sheath, it may track back to the brainstem and cause brainstem anaesthesia. The patient may stop breathing and lose consciousness, and this may be accompanied by fitting (epileptic seizures) and unstable blood pressure. Death is likely to occur if these complications are not managed properly. If the needle pierces an artery, this may cause a tense haematoma (retrobulbar haemorrhage): if not managed properly, this could cause globe ischaemia and blindness. Adrenaline (epinephrine) in the anaesthetic mixture could cause vasospasm, and this has been implicated as a cause for ‘wipe-out’ syndrome: postoperative blindness with no obvious cause.

## Sub-Tenon's

Sub-Tenon's blocks are thought to be much safer than sharp-needle blocks. However, all of the above complications could still occur. If a long metal cannula is used, this might perforate the optic nerve sheath or the arteries at the back of the globe. If these arteries are damaged, blindness appears more likely to occur from ischaemia, rather than due to pressure from a haematoma. The sub-Tenon's technique requires some dissection of the conjunctiva and Tenon's capsule in order to reach the sub-Tenon's space. If there isa lot of scar tissue (e.g. from previous surgery, injury, traditional medicines, etc.) this may result in an inadvertent globe perforation. The sub-Tenon's dissection also provides a possible route for bacteria to enter, with the potential for an orbital infection.

**Figure:**
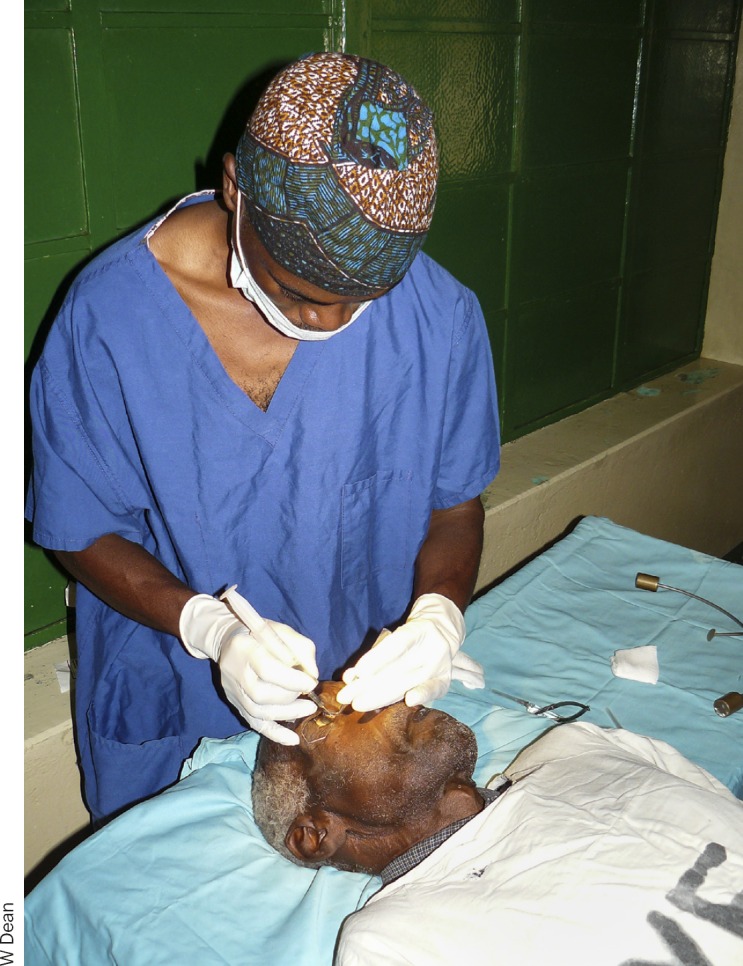
Sub-Tenon's local anaesthesia

## Minimal anaesthesia (topical, intra-cameral, sub-conjunctival)

With minimal anaesthesia techniques, these risks are largely avoided, but these techniques have other potential problems. Retrobulbar, peribulbar and sub-Tenon's local anaesthesia will temporarily paralyse the extra-ocular muscles, providing good operating conditions with an immobile eye. By contrast, topical, intra-cameral and sub-conjunctival local anaesthesia result in a potentially ‘mobile’ eye, and this might lead to challenges for the surgeon and possibly complications and a poor outcome. Many surgeons believe that these techniques are unsuitable for surgery on the ‘open eye’ (e.g conventional sutured large Incision extra-capsular cataract extraction), because squeezing of the extra-ocular muscles may increase the risk of vitreous loss, choroidal haemorrhage, ora blinding expulsive haemorrhage. These ‘minimal’ techniques are also usually unsuitable for manual small incision cataract surgery (SICS). Thus, there are many situations in which a ‘block’ – either a needle block or sub-Tenon's block – is preferable.

## Reducing the risks

There are several ways to reduce the risks from local anaesthesia in the eye. Of course, anyone giving local anaesthesia should have appropriate training and an understanding of the relevant anatomy. The anaesthesia/surgical area should be adequately sterile, and there should be sufficient personnel with the right skills for safe local anaesthesia and surgery. There should be an agreed method for monitoring patients during surgery, and an agreed protocol to get help if resuscitation is required.

‘Default’ local anaesthesia techniques should be chosen, appropriate for your population and the type of surgery you do: for example, pterygium surgery using sub-conjunctival local anaesthesia (given by the surgeon after topical anaesthesia), large-incision cataract surgery using sub-Tenon's block (given 15 minutes before surgery). All patients should be assessed for problems that could make local anaesthesia more risky or might suggest an alternative technique, e.g. abnormal globe, abnormal globe position and/or conjunctival scarring.

Myopic (near-sighted) eyes are larger and longer than normal, and may also have a posterior staphyloma, which means that local anaesthesia delivered by a needle will be more likely to perforate the globe. Biometry can be helpful, if available: myopic eyes with an axial length over 26 mm have a significantly higher risk of globe perforation from needle blocks using an infero-temporal approach. Before giving the anaesthetic, checks should be done to ensure that the correct patient has the correct operation, on the correct eye. The fornices should be cleaned with povidone iodine 5% to minimise the risk of infection. Aseptic techniques should always be used.

## Recommended approaches

### Sub-Tenon's block

Sub-Tenon's block provides an excellent balance of risks and benefits for the majority of intraocular surgery.

Disposable sub-Tenon's cannulae are available, although other cannulae can also be used to minimise expense. Many practitioners have had success with plastic intravenous cannulae, metal lacrimal cannulae, or 22-gauge Rycroft cannulae (e.g the cannulae that are often provided with hydroxypolymethylcellulose [HPMC]). Whichever cannula is used, it is important to avoid pushing the cannula too far back, because of possible damage to the optic nerve or the arteries at the back of the globe (the short posterior ciliary arteries). Of course, if an intravenous cannula is used for sub-Tenon's LA, the sharp needle should be removed first.

### Technique for sub-Tenon's LA

A syringe is prepared with anaesthetic and a blunt-ended sub-Tenon's cannula (see previous paragraph). If the surgeon requires anaesthesia without akinesia, 2 ml of lidocaine (lignocaine) will be adequate. If the surgeon requires akinesia, then larger volumes and/or additional hyaluronidase may be used.

**Figure 1:**
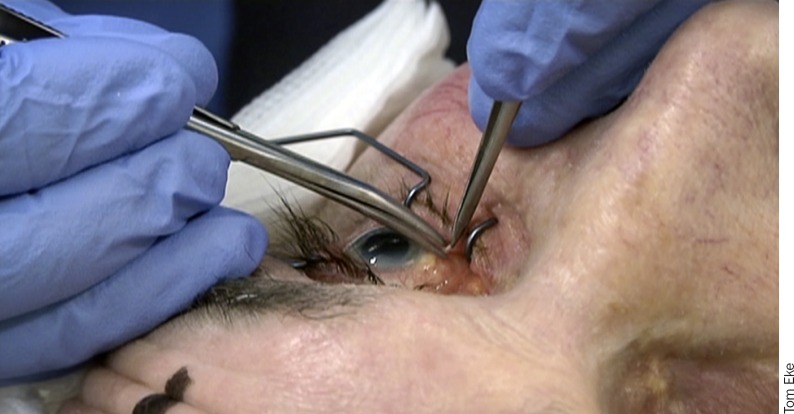


**Figure 2:**
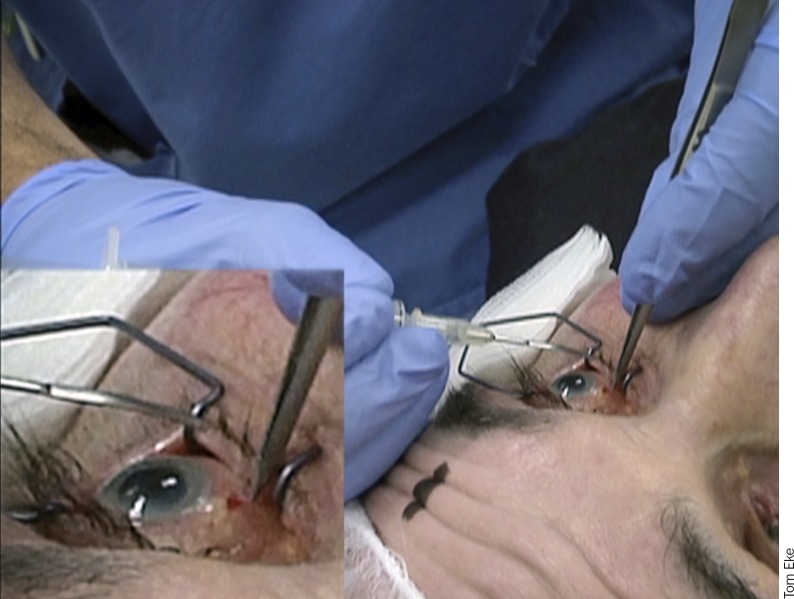


**Figure 3:**
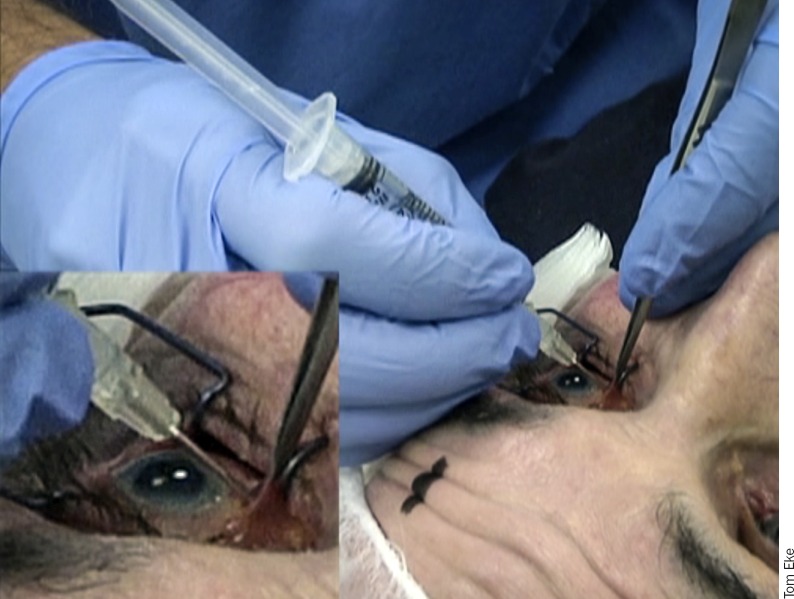


1. Instil topical anaesthesia (e.g. lidocaine or proxymetacaine) and povidone iodine.
2. Insert a lid speculum or hold the eyelids open with the fingers.
3. Tell the patient to look ‘up and out’ (i.e up towards the eyebrows, and out toward the ear on that same side). This will expose the conjunctiva in the infero-nasal part of the globe.
4. Grasp the conjunctiva with conjunctival forceps, about 5 mm posterior to the limbus, between the insertions of the medial rectus and inferior rectus (Figure 1).
5. Elevate the conjunctiva slightly, and use spring-scissors to make a small ‘snip’ in the conjunctiva and the underlying Tenon's capsule (Figure 2). When positioned 5–6 mm behind the limbus, a tiny snip (about 2–3 mm) will usually go through both layers at once and expose the shiny white sciera below. If the snip is too small or too superficial to expose the sciera, it may be necessary make another snip to enlarge the hole. Alternatively, it may be necessary to make a small dissection by inserting the closed scissors into the incision, then opening the scissors to spread the tissues. Do this with the scissors positioned perpendicular to the globe, in order to dissect down on to the bare sciera, 5-6 mm from the limbus.
6. Hold the posterior edge of the incision with forceps and introduce the sub-Tenon's cannula (Figure 3).
7. Advance the cannula posteriorly (tangential to the globe) with a sweeping motion, so that the cannula tip remains in contact with the sciera until it is just behind the equator of the globe. It is usually possible to feel that the cannula tip remains in contact with the smooth sciera as you advance the cannula.
8. Slowly inject the anaesthetic and then remove the cannula.
9. There is usually no need for a pressure device (weight), but is it important to ensure that the lids remain closed so that the cornea does not become dry.

**Figures 1–3: Sub-Tenon's LA using a 19-gauge ‘Stevens’ angled metal cannula**

**Figure:**
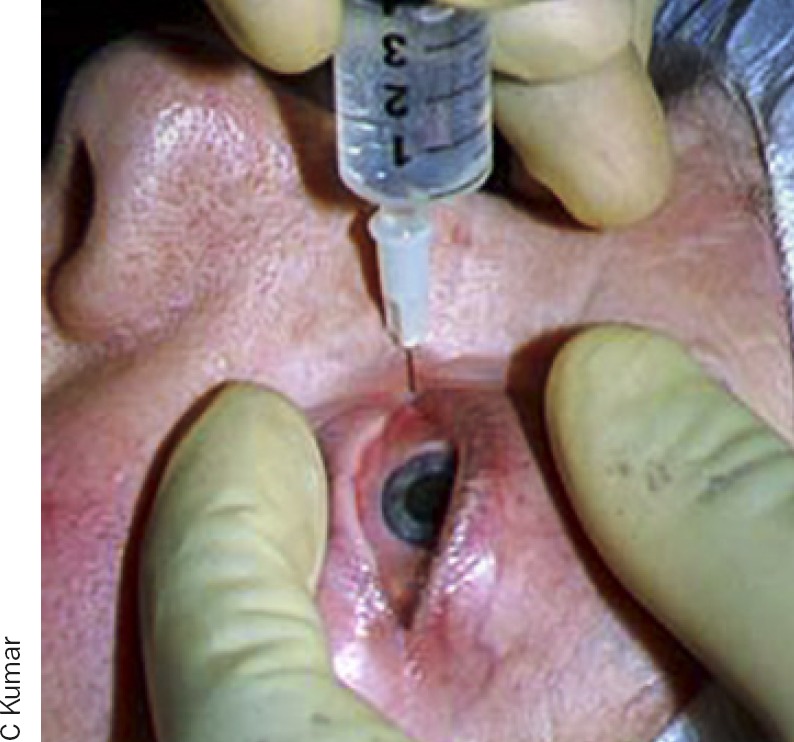
Single medial peribulbar block

### Single medial peribulbar block

If using a sharp-needle technique, the safest approach appears to be the single medial peribulbar block. With this approach, the needle is less likely to perforate the globe or cause a retrobulbar haemorrhage or brainstem anaesthesia, but these complications are still possible with this technique. Instead of the classic infero-temporal approach, the needle is inserted to the medial part of the orbit, through the lacrimal caruncle.

### Technique for medial peribulbar block

1. Ask the patient to lie on her or his back, facing the ceiling with the eyes in the primary gaze (looking straight ahead)
2. Instil local anaesthetic and iodine drops
3. Prepare the syringe, using a short needle (e.g. standard hypodermic needle, 25 mm or shorter, narrow gauge, e.g. 25 gauge).
4. Position the syringe so that the needle is pointing vertically downward towards the floor. The aim is to insert the needle into the medial part of the orbit, near the medial canthus (where the two lids meet, near the nose).
5. Use the needle to pierce the lacrimal caruncle, as far medially as possible. Direct the needle ‘backwards’ along the medial wall of the orbit, i.e. if the patient is lying down and facing the ceiling, then you should advance the needle towards the floor of the room.
6. Inject the anaesthetic slowly, and then withdraw the needle.
7. Hold the eyelids closed to prevent corneal drying. Intermittent pressure may aid dispersal of the anaesthetic.

## Complications

The surgical/anaesthesia team should be aware of the most serious complications of local anaesthesia and take steps to minimise the risk. It is also important to know what to do, should a serious complication occur.

### Brainstem anaesthesia

The signs are variable, but the patient is likely to become drowsy or lose consciousness, within seconds or minutes of the anaesthetic being given. This rare complication is more likely to occur with sharp-needle techniques, but can still occur with sub-Tenon's. Blood pressure may be high, then low, the patient may stop breathing and epileptic seizures may occur. It is important to have a plan for when this occurs: resuscitation should be commenced, and ideally an anaesthetist should be available to manage the airway and monitor the patient in an intensive care setting. With proper support, the patient should recover when the anaesthetic wears off. It is best to try to minimise the risk of brainstem anaesthesia by following the recommendations in this article.

### Retrobulbar haemorrhage

This presents with a tense, hard orbit, usually within seconds or minutes of LA being given. Again, this complication is more likely to occur with sharp-needle techniques. The surgeon should be informed immediately, because the high orbital pressure may cause globe ischaemia and blindness. Making a cut in the lateral canthus (lateral canthotomy) may decompress the orbit sufficiently with a cut in the skin from the lateral canthus to the lateral bony wall of the orbit. Often it is necessary to make a second cut, at the lateral end of this first cut, directed down toward the infero-temporal corner of the orbital margin (lateral cantholysis). This should allow the orbital pressure to lower, thus saving the sight. The lid can then be repaired after a day or two.

### Globe perforation

This can be more difficult to manage. Some cases may resolve spontaneously, but severe scierai lacerations, choroidal haemorrhage, and/or retinal detachment is likely to need vitreoretinal surgery. Patients with globe perforation should be seen by a vitreoretinal surgeon as soon as possible.

**Useful resources**

For more information on local anaesthesia for eye surgery, numerous resources are available. All of the references and links below are freely available from the internet. The guideline ‘Local anaesthesia for ophthalmic surgery’ was written with UK practice in mind, but most of the recommendations are applicable worldwide and there is a section on ‘Local anaesthesia complications and how to avoid them’. Regular conferences on eye anaesthesia take place in the USA, UK and India, and the World Congress of Ophthalmic Anaesthesia will take place in Indonesia in 2020.

**Guidelines**

KumarCMEkeTDoddsC et al. Local anaesthesia for ophthalmic surgery (guideline).
London: Royal College of Anaesthetists, Royal College of Ophthalmologists, 2012
**https://www.rcophth.ac.uk/wp-content/uploads/2014/12/2012-SCI-247-Local-Anaesthesia-in-Ophthalmic-Surgery-2012.pdf**10.1038/eye.2012.82PMC337630422538216BenjaminLAllenBDesaiR et al. Cataract Surgery Guidelines September
2010
London: Royal College of Ophthalmologists, 2010. **https://www.rcophth.ac.uk/wp-content/uploads/2014/12/2010-SCI-069-Cataract-Surgery-Guidelines-2010-SEPTEMBER-2010.pdf**

**Practical techniques (all references include illustrations of techniques)**

GuiseP
Sub-Tenon's anaesthesia.
Local & Regional Anaesthesia
2012:5; 35–46.10.2147/LRA.S16314PMC341798022915900KumarCMDoddsC
Ophthalmic regional block.
AnnAcad Med Singapore
2006; 35:158–167.16625264BurkatCLemkeB
Retrobulbar hemorrhage: anterolaterateral anterior orbitotomy for emergent management.
Arch Ophthal
2005;123:1260–1262
**http://archopht.jamanetwork.com/article.aspx?articleid=417269**1615780910.1001/archopht.123.9.1260BallardSR et al. Emergency lateral canthotomy and cantholysis: a simple procedure to preserve vision from sight threatening orbital hemorrhage.
J Spec Operations Med
2009;9:26–31
**www.jsomonline.org/Publications/2009326Ballard.pdf**10.55460/1CLD-XJUV19739474

**Websites**

British Ophthalmic Anaesthesia Society**www.boas.org**Ophthalmic Faculty of the Indian Society of Anaesthesiologists**www.ofisa.sankaranethralaya.org**Ophthalmic Anesthesia Society (USA)**www.eyeanesthesia.org**
